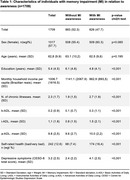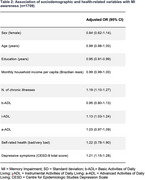# Awareness of Memory Impairment in a representative sample of Brazilians aged 50 years and over (ELSI‐Brazil)

**DOI:** 10.1002/alz70857_107135

**Published:** 2025-12-26

**Authors:** Pedro JDMR Pinho, Laiss Bertola, Daniel C. Mograbi, Lucas Martins Teixeir, Matheus Ghossain Barbosa, Maria Fernanda Lima‐Costa, Cleusa P Ferri

**Affiliations:** ^1^ Universidade Federal de São Paulo (UNIFESP), São Paulo, São Paulo/SP, Brazil; ^2^ Pontifical Catholic University of Rio de Janeiro, Rio de Janeiro, Brazil; ^3^ Fiocruz, Belo Horizonte, MG, Brazil; ^4^ Hospital Alemão Oswaldo Cruz, São Paulo, São Paulo, Brazil

## Abstract

**Background:**

Memory impairment (MI) is one of the most common and early symptoms of cognitive decline and dementia. However, individuals with MI can be unaware of their condition. This occurrence is still understudied in socially and ethnically diverse populations, such as Brazilians.

**Method:**

Secondary analysis of baseline data from the ELSI‐ Brazil study, representative sample of Brazilians aged 50 years and over, with 9402 individuals.

Memory impairment (MI) was defined through a z‐score reflecting how many standard deviations the participant deviates from a predicted score for them based on their age, education, and gender/sex derived of combined immediate and delayed response from the 10‐word list derived from a healthy subset of individuals from the ELSI‐Brazil. Awareness was assessed with the question: “Currently, how do you rate your memory”, and categorized as with awareness (answers fair or bad) and without awareness (answers excellent, very good or good).

Other measures included sociodemographic factors (sex, age, education, household income), number of depressive symptoms (CESD‐8), self‐rated health, number of chronic illnesses, and activities of daily living: basic (b‐ADL), instrumental (i‐ADL) and advanced (a‐ADL).

Logistic regression with robust variance was used to estimate the association between the variables above with awareness of MI.

**Result:**

From the total original sample (*n* = 9412), 1709 individuals (21.7%) had MI and the majority were women (57.7%), with a mean age of 62.8 years (SD = 9.9) and an average of 5.4 years of education (SD = 4.5). Nearly half (*n* = 826) of individuals with MI were aware of their MI.

Those with smaller years of education, higher number of chronic illnesses, higher disability in i‐ADL and higher number of depressive symptoms were more likely to be aware of their memory impairment. Sex, age, income, disability in b‐ADL and a‐ADL, and self‐rated health were not associated with MI awareness.

**Conclusion:**

Less than half of individuals with MI were aware of their condition, and those with other health and functionality problems were more likely to be aware of their MI. Higher educational attainment, better health and functioning may be related to higher cognitive reserve and may mitigate individual's ability to self‐perceive their MI.